# Regenerative potential of primary adult human neural stem cells on micropatterned bio-implants boosts motor recovery

**DOI:** 10.1186/s13287-017-0702-3

**Published:** 2017-11-07

**Authors:** Carole Davoust, Benjamin Plas, Amélie Béduer, Boris Demain, Anne-Sophie Salabert, Jean Christophe Sol, Christophe Vieu, Laurence Vaysse, Isabelle Loubinoux

**Affiliations:** 1 ToNIC, Toulouse NeuroImaging Center, Université de Toulouse, Inserm, UPS, Toulouse, France; 2 LAAS-CNRS, Université de Toulouse, CNRS, INSA, UPS, Toulouse, France; 3 Centre Hospitalier Universitaire de Toulouse, Pôle Neurosciences, CHU Toulouse, Toulouse, France; 4 UMR1214—Inserm/UPS—ToNIC, CHU PURPAN, Pavillon Baudot, Place du Dr Baylac, 31024 Toulouse cedex 3, France

**Keywords:** Brain injury, Cell therapy, Biomaterial, Tissue engineering, Sensorimotor recovery

## Abstract

**Background:**

The adult brain is unable to regenerate itself sufficiently after large injuries. Therefore, hopes rely on therapies using neural stem cell or biomaterial transplantation to sustain brain reconstruction. The aim of the present study was to evaluate the improvement in sensorimotor recovery brought about by human primary adult neural stem cells (hNSCs) in combination with bio-implants.

**Methods:**

hNSCs were pre-seeded on implants micropatterned for neurite guidance and inserted intracerebrally 2 weeks after a primary motor cortex lesion in rats. Long-term behaviour was significantly improved after hNSC implants versus cell engraftment in the grip strength test. MRI and immunohistological studies were conducted to elucidate the underlying mechanisms of neuro-implant integration.

**Results:**

hNSC implants promoted tissue reconstruction and limited hemispheric atrophy and glial scar expansion. After 3 months, grafted hNSCs were detected on implants and expressed mature neuronal markers (NeuN, MAP2, SMI312). They also migrated over a short distance to the reconstructed tissues and to the peri-lesional tissues, where 26% integrated as mature neurons. Newly formed host neural progenitors (nestin, DCX) colonized the implants, notably in the presence of hNSCs, and participated in tissue reconstruction. The microstructured bio-implants sustained the guided maturation of both grafted hNSCs and endogenous progenitors.

**Conclusions:**

These immunohistological results are coherent with and could explain the late improvement observed in sensorimotor recovery. These findings provide novel insights into the regenerative potential of primary adult hNSCs combined with microstructured implants.

## Background

The increasing number of neurological injuries over recent years has become a major public health problem. Cellular and neuronal loss may occur after traumatic brain injury, subarachnoid haemorrhage or stroke, leading to serious and sometimes fatal deficits. At present, none of the current available treatments allow for complete functional recovery in severely impaired patients with acquired deficits.

The adult brain still contains neural stem cells (NSCs), particularly in zones known as neurogenic niches [1]. In humans, the main neurogenic niches are the subventricular zone (SVZ) and the subgranular zone (SGZ) in the dentate gyrus (DG) of the hippocampus [2]. These adult NSCs are capable of self-renewal [3], can generate the three main lineages of the brain (neurons, astrocytes and oligodendrocytes) [4] and have limited proliferation [5].

Interestingly, after a brain insult, adult neurogenesis is boosted [6] and NSCs are attracted to the damaged area [7, 8] up to 1 year after the infarct [9]. Nonetheless, most of the newly formed progenitors fail to survive in the long term [10, 11] or to differentiate into mature cells [12]. Because the brain is unable to sufficiently regenerate itself, one potential solution is to develop cell replacement strategies. Several cell sources are potentially available for graft, including neural cell lines [13], embryonic stem cells [14], mesenchymal stem cells (MSCs) [15] and induced pluripotent stem (iPS) cells [16]. However, the first two sources raise ethical concerns, while the potential of the other two sources to differentiate into neuronal cells is still very low. Mounting evidence shows the potential role of adult hNSCs in brain tissue regeneration after a cerebral lesion, even in humans [7]. To study their potential, we chose to graft adult hNSCs. At present, the best sources of neural stem/progenitor cells in the adult brain are human biopsies of the SVZ and temporal lobe [17–19].

Once grafted, NSCs can contribute to the tissular reconstruction. They are also known to release neurotrophic and immunoprotective factors that promote the restoration of brain tissue and prevent the damage from spreading [20]. Whatever the cell source, motor recovery is not complete in animal models [21], essentially owing to poor engraftment [22]. Tissue engineering could provide solutions in the form of 3D, to improve cell survival and differentiation [23]. Maturation of grafted cells has been demonstrated to take months [24]. Therefore, non-degradable biomaterials have to be tested first to guarantee optimal neuronal differentiation. Biomaterials have been developed to locally deliver pharmacological growth factors such as vascular endothelial growth factor (VEGF) [15] and brain-derived neurotrophic factor (BDNF) [25] to favour tissue reconstruction. Another approach consists of seeding cells, be they adult rat neural progenitor cells [26], human MSCs [22] or hNT2, a human neuronal cell line [27], on the scaffold before implantation, to enhance the potential effect of both grafted cells and biomaterials [27]. The challenge is still to find both a biomaterial adapted to neuronal differentiation and cell sources able to regenerate adult human brain in order to restore sensorimotor functions.

We have developed a new micropatterned polydimethylsiloxane (PDMS) device for brain tissue reconstruction. The design of these scaffolds and their dimensions have been optimized to allow for neuronal development and hNSC differentiation, and to align neurites along microchannels, thus favouring long-axon formation [28]. A pilot study has shown the feasibility of inserting these PDMS scaffolds combined with a neuronal cell line in a model of cortico-striatal injury [29].

In the present report, we assessed motor recovery following a cortico-striatal bio-prosthesis consisting of a graft of primary hNSCs isolated from patient biopsies, pre-seeded on 3D micropatterned implants. The so-called neuro-implants were directly inserted dorso-ventrally in the infarct area 2 weeks after injury in a rat model. Cell integration, neuronal differentiation, cell migration and glial reaction were particularly studied using immunohistological approaches.

## Methods

### hNSC-micropatterned bio-implant preparation

#### Fabrication of 3D micropatterned PDMS implant

The implantable device, already described elsewhere [28, 30], consisted of two PDMS micropatterned layers assembled (300 μm thick) to form a 3D microstructured implant (2 mm × 4 mm). PDMS shows excellent resistance to biodegradation and ageing, high biocompatibility and has United States Pharmacopeia (USP) class VI clinical approval for unrestricted use in chronic implants [31]. The micropattern was an array of linear microchannels, 25 μm deep, with a groove width of 60 μm and a terrace width of 10 μm, which allowed directing axonal and neurite growth without disturbing cell differentiation. To favour cell adhesion, these PDMS substrates were treated in O_2_ plasma for 2 min and then coated with polylysine (100 μg/ml; Sigma-Aldrich) and laminin (40 μg/ml; Sigma-Aldrich).

#### hNSC cell culture and seeding on 3D micropatterned surface

Biopsies from the temporal lobe and SVZ were obtained from individuals undergoing neurosurgery for epilepsy treatment (*N* = 10). All procedures were performed with informed patient consent, authorized by our local human ethics committee (Comité de Protection des Personnes Sud-Ouest et Outre Mer Toulouse I) in accordance with institutional guidelines on human tissue handling and use. To isolate potential neural progenitor/stem cells, cell suspensions were rapidly obtained by tissue enzymatic digestion and amplified as neurospheres, as already described [18].

At the end of the amplification phase, the neurospheres were dissociated, to obtain single-cell suspensions, and used for direct cell graft or for cell seeding on the 3D micropatterned implants [29]. hNSCs were seeded at a final density of 125,000 cells/cm^2^ on one side of the PDMS, and the day after on the other side, representing 20,000 cells per implant. To obtain the final neural implant, cells were cultured for 3 days before implantation, with NGF (20 ng/ml; Peprotech) to favour neuronal differentiation [18].

### Animals, M1 lesion induction and implantation

All of the animals (*N* = 61) were maintained and treated according to Council of the European Communities guidelines (Directive of 24 November 1986, 86/609/EEC). This protocol was approved by the Direction départementale de la Protection des Populations de la Haute-Garonne (authorization no. 31125507) and the Comité d’éthique pour l’expérimentation animale Midi-Pyrénées. All efforts were made to minimize the number of animals used and to avoid suffering.

Adult female (300–350 g) Sprague–Dawley rats (Elevage Janvier, Le Genest-St-Isle, France) were anaesthetized with ketamine/medetomidine (36/0.47 mg/kg, intraperitoneal injection) and pre-medicated with long-acting oxytetracycline (60 mg/kg) and methylprednisolone (0.4 mg/kg).

**Fig. 1:**
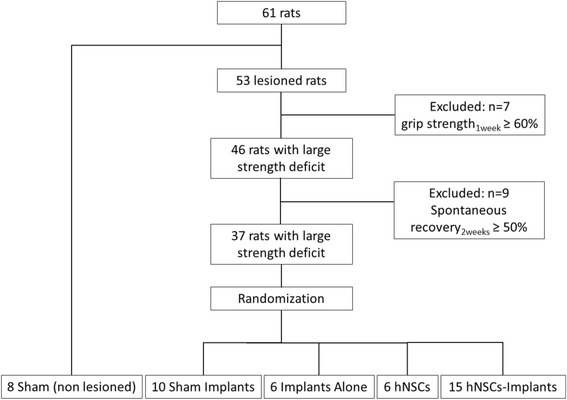
Flowchart of the study. hNSCs human neural stem cells

Cortical lesions focused on the caudal forelimb motor area (M1) were induced by malonate injection (5 μl, 3M solution, pH 7.4 in PBS; Sigma-Aldrich, France) (*n* = 53) or PBS for the sham group (*n* = 8) [27] at the following stereotaxic coordinates: 2.5 mm lateral to Bregma, and 2 mm deep. Only animals that displayed substantial neurological dysfunctions (grip strength < 60% of pre-lesion value, *n* = 46 out of 53 lesioned rats) 1 week after injury were selected (Fig. 1). The animals that showed excessive spontaneous recovery in 1 week (grip strength improvement in 1 week ≥ 50% of post-lesion value) were then discarded (*n* = 9 out of 46). Finally, 37 rats were randomly assigned to the different treated groups.

Two weeks after the lesion, a second surgical intervention for the implantation was then performed under inhalational anaesthesia with 1.5% isoflurane (Virbac, France) in 100% O_2_. A group of lesioned rats each received five implants pre-seeded with a total of 100,000 hNSCs in the lesion (hNSC Implants group, *n* = 15). Another group of lesioned animals each received five implants without any cells (Implants Alone group, *n* = 6), and another group underwent the same surgery but the implants were immediately removed (Sham Implants group, *n* = 10). The final group of lesioned rats received a cell graft of 5 × 10^5^ hNSCs, by perfusion of 5 μl of cell suspension at 1 μl/min (hNSCs group, *n* = 6), as already described elsewhere [27].

### Grip strength test

Before the study, the animals were acclimatized and trained on a controlled diet for the behavioural tests, as already described [27]. Behavioural testing to detect motor deficits was performed 1 week before lesion (baseline), then 48 h post lesion, 24 h post graft and once a month for 3 months after the insult. Experimenters were blind to the treatment group.

All of the rats underwent strength testing (3 trials/paw/day) with the grip strength test, on 3 consecutive days, and a mean score was calculated for each of the six different time points. Post-lesional performances of the contralesional forelimb were expressed as a percentage of the pre-lesional baseline value. Each value reported here represents the median ± interquartile range (first quartile (Q1); third quartile (Q3)) of each group.

### Post-mortem MRI and global atrophy measurement

Either the whole animal anaesthetized or just the brain was scanned on a 3T Achieva (Phillips) MRI scanner. Horizontal and coronal T2-weighted images were acquired (TR 2 s, TE 81 ms, impulsion angle 90°, FOV 200 mm, matrix 176 × 150, voxel size 0.34 mm × 0.34 m × 0.8 mm).

Hemispheric volume was determined using MRIcro on the horizontal slices. Hemispheric atrophy was then calculated using the following formula:

$$ \mathrm{Atrophy}\ \left(\%\right)=\frac{\left(\mathrm{Healthy}\  \mathrm{hemisphere}\  \mathrm{volume}\hbox{-} \mathrm{Lesioned}\  \mathrm{hemisphere}\  \mathrm{volume}\right)}{\mathrm{Healthy}\  \mathrm{hemisphere}\  \mathrm{volume}}\times 100 $$

### Tissue processing, fluorescence immunolabelling and quantification

#### Tissue processing

After induction of anaesthesia, rats were perfused intracardially with 4% paraformaldehyde solution. The brains were then embedded in 3% low gelling temperature agarose (Sigma-Aldrich). To preserve biomaterials, thick horizontal brain sections (500 μm) were cut on a vibratome. For fluorescence immunolabelling, section levels were identified using the Paxinos and Watson [32] atlas, after binocular observations. Sections of the same brain level were subsequently taken for each marker in all animals. Free-floating sections were permeabilized with 0.1% Triton X-100 (Sigma-Aldrich) in PB for 40 min at room temperature, and incubated with blocking solution (3% goat serum). Sections were then allowed to react with primary antibodies for 48 h at 4 °C, using the appropriate dilution: goat anti-nestin 1:300 (SantaCruz); goat anti-DCX 1:200 (SantaCruz); mouse anti-Tuj1 1:500 (neuron-specific class III beta-tubulin; Covance); rabbit anti-Tuj1 1:300 (Covance); rabbit anti-MAP2 1:200 (microtubule associated protein 2; Millipore); mouse anti-SMI312 1:500 (Abcam); rabbit anti-NeuN 1:300 (neuronal nuclear antigen; Abcam); rabbit anti-GFAP 1:1000 (glial fibrillary acidic protein; DAKO); and rabbit anti-CD68 1:300 (ED1; Millipore). Detection and characterization of the grafted hNSCs were performed using the mouse anti-hMTCO2 1:300 (Abcam) or mouse anti-hNCAM 1:200 (SantaCruz) human markers, in combination with another primary antibody (as specified). After PB washes, the appropriate secondary antibody (Alexa Fluor 488 or 568; Molecular Probes) was incubated for 24 h at 4 °C. Nuclei were stained with DAPI (0.25 μg/ml; Sigma-Aldrich). Negative controls were performed for all immunostainings. The specificity of the two anti-human antibodies has been checked in our experiments by including sections, run in parallel, from a control group without grafted cells.

#### Image acquisition and quantification

Brightfield and fluorescence images were captured using a fluorescence stereo-microscope (Axiozoom V16; Zeiss) and a confocal microscope (LSM 710; Zeiss) for zoomed images.

To analyse the injured area, brightfield images of the whole-brain sections were examined. The reconstruction percentage, essentially corresponding to implants together with the newly generated tissue, was measured as follows. First, a volume called the total reorganized volume and corresponding to the lesion cavity, newly reorganized tissue, implants and dilated ventricle was estimated using ImageJ. To better estimate the initial lesion volume, the mean volume of normal ventricles derived from six healthy brains (1.61 ± 0.54 mm) was subtracted. A third volume, the cavity volume, including the lesion cavity and the dilated ventricle was evaluated. The reconstruction percentage for each group was then calculated as follows:

$$ \mathrm{Reconstruction}\  \mathrm{percentage}=\frac{\left(\mathrm{Total}\  \mathrm{reorganized}\  \mathrm{volume}\hbox{-} \mathrm{Cavity}\  \mathrm{volume}\right)}{\left(\mathrm{Total}\  \mathrm{reorganized}\  \mathrm{volume}-\mathrm{Normal}\  \mathrm{ventricle}\  \mathrm{volume}\right)}\times 100 $$

Immunostaining quantifications were performed with MorphoStrider (ExploraNova, France) on images taken with the same parameters for each staining. Given the thickness of the sections, quantification of surviving cells was not possible.

For ED1 and GFAP expression analyses, signals were assessed on four independent fields (2.3×) surrounding the lesion on four sections per animal and five different animals per group. The presence of activated microglia was expressed as the number of ED1-positive cells/mm^2^ of analysed tissue and the astroglial signal was expressed as GFAP-positive surface/mm^2^ of analysed tissue.

To estimate the level of grafted cell differentiation, single-stained and double-stained cells were counted using ImageJ (cell counter plugin) on Axiozoom images (2.3×) for implants or on confocal images (63×) for tissue analyses. Counting was performed on five independent fields per animal, and expressed as a percentage of differentiation.

### Statistics

Data were analysed with a linear mixed effect model and function using the R software (3.3.3 version) [33]. Time and groups were fixed as factors and interaction between time and group was analysed in the ANOVA. Each rat was considered as a random factor. Post-hoc contrast analyses were done at each time using the least square means approach (MASS and lsmeans package, function contr.sdif) [34]. All other data (immunostaining quantification) were analysed using GraphPad Prism v. 6.01 software and the non-parametric Kruskal–Wallis test followed by Dunn’s test.

## Results

### Long-term strength improvements with neuro-implants

**Fig. 2:**
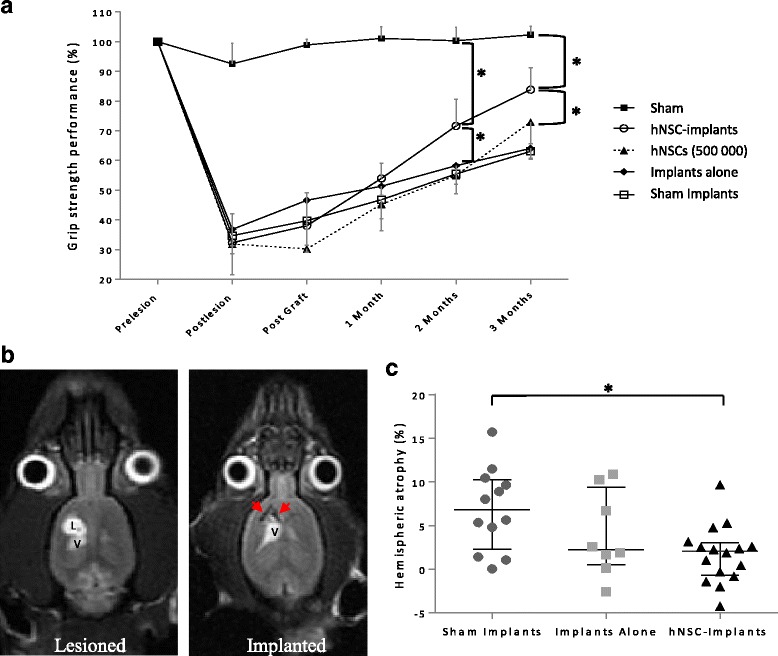
Functional recovery and evaluation of hemispheric atrophy with neuro-implants inserted in a brain lesion. **a** Grip strength performance at different time points after transplantation of neuro-implants pre-seeded with hNSCs (hNSC Implants group, *n* = 15), or implants on their own (Implants Alone group, *n* = 8), or direct engraftment of a neural cell suspension (hNSCs group, 500,000 cells, *n* = 6). Controls were sham-operated for the lesion (Sham group, *n* = 8) or implantation (Sham Implants group, *n* = 10). Statistical analysis carried out with a linear mixed effect model (significant differences reported on the graph at 2 and 3 months, *p < 0.05). **b** Representative horizontal T2-weighted MRI images in a Sham Implants (Lesioned) rat and an Implants Alone (Implanted) rat. L lesion, V ventricle. Red arrows indicate PDMS implants. **c** Percentage of atrophy of the lesioned hemisphere in each group, as assessed on T2 MRI images. Data represent individual values and median ± interquartile range. Statistical analysis carried out with the Kruskal–Wallis test followed by Dunn’s test. *p < 0.05. hNSC human neural stem cell

The cortico-striatal lesion induced motor deficit of the contralesional paw. The grip test provides a sensitive quantitative measure of the forelimb (Fig. 2a). Post-lesional performances of the contralesional forepaw were very similar across all the lesioned groups, falling to around 34.2% (28.0; 42.0). There was a highly significant time effect, group effect and interaction between time and group (*p* < 10^–6^). Comparison with the hNSC rats showed that the hNSCs Implants group significantly improved spontaneous recovery from the first month onwards (*p* < 0.05), and then at 2 and 3 months (*p* < 0.002 and *p* < 0.05) with a maximum median performance of 83.8% (79.7; 90.9). Nevertheless, they remained significantly different from the Sham-lesioned rats (*p* < 0.0001). By contrast, the Implants Alone group at 3 months did not differ statistically from either the Sham Implants group (*p* = 0.46) nor the hNSCs group (*p* = 0.33). This result suggests that the presence of the implant in the lesion cavity did not have any deleterious long-term effect on spontaneous recovery. Even so, a direct graft of 500,000 hNSCs was unable to enhance the recovery kinetics compared with the hNSC Implants group with only 100,000 cells. These data suggest that the combination of the implants with the hNSCs is needed to bring about a therapeutic effect.

### Hemispheric atrophy reduction and tissue reconstruction after neuro-implant engraftment

**Fig. 3:**
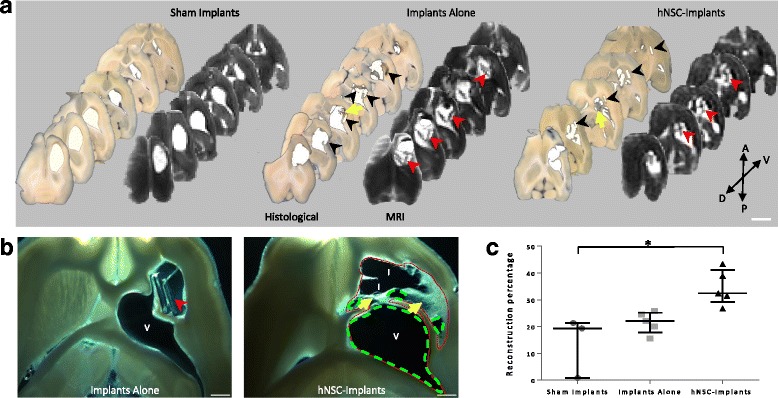
Effect of PDMS implants on tissue reconstruction 3 months after lesion. **a** Comparison between horizontal, histological and MRI slices in a representative rat of each group (lesioned area in white). Implants visible on MRI slices (red arrowheads). Most of the time, only the position of the implant was observable on the histological slices (black arrowheads), surrounded by newly generated tissue (yellow arrows). A–P antero-posterior axis, D–V dorso-ventral axis. Scale bar: 500 μm. **b** Representative horizontal brain section of the lesioned area under brightfield illumination from Implants Alone (left) and hNSC Implants (right) rats. Newly generated tissue (yellow arrow) was mostly located around the PDMS implants which could still be found in place in the lesion core after cutting (red arrowhead). V ventricle, I implant position. Red ROI indicates total volume (lesion cavity, dilated ventricle, newly reorganized tissue around implants). Green ROI indicates cavity volume (lesion cavity and dilated ventricle). Scale bar: 1 mm. **c** Percentage of reconstruction was calculated on brightfield images in each group. Data represent individual value and median ± interquartile range. Statistical analysis carried out with the Kruskal–Wallis test followed by Dunn’s test. *p < 0.05. hNSC human neural stem cell, MRI magnetic resonance imaging

Injection of malonate led to lesions that induced a cavity at 3 months on T2-weighted MRI and a dilation of the ipsilesional lateral ventricle both hyperintense on T2 images at 3 months (Fig. 2b, Lesioned). In addition, measures of the two hemispheric volumes showed that injury was accompanied by atrophy of 6.8% (3.9, 9.8) at 3 months for the lesioned group (Sham Implants, Fig. 2c). In the implanted groups, T2 images confirmed that the implants, apparent as black rectangles, were still located at the core of the lesion after 3 months (Fig. 2b, Implanted). The presence of implants limited atrophy and dilation of ventricle. Atrophy was non-significantly decreased to 2.2% (1.3; 7.6) for the Implants Alone group and significantly to 2.1% (–0.4; 2.7) for the hNSC Implants group compared with the Sham Implants group (*p* < 0.05; Fig. 2c). The collapse of brain tissue was reduced by a mechanical effect of PDMS devices and brain morphology tended to be preserved. To further analyse the impact of the implants on tissue regeneration, histological brain sections were performed. Sections 500 μm thick were a good compromise between keeping the implants in place as often as possible during the cutting and carrying out the immunostaining. Analysis of whole sections by light microscopy demonstrated a consistent matching with MR images for the lesion cavity and dilated ventricle (Fig. 3a). Dilation of the ventricle was impressive in the Sham Implants group. In contrast to the MRI slices, the implants were not always directly observable, but left a rectangular print that was visible on these brightfield sections and was surrounded by newly regenerated tissue, notably in the hNSC Implants group (Fig. 3b). In the Sham Implants group, the newly generated tissue represented just 19.3% (10.1; 20.3) of the reorganized volume, so reconstruction was limited 3 months post lesion. The Implants Alone group had the same profile, with 22.1% (20.0; 24.5) of reconstruction, suggesting that simply inserting implants in the core lesion is not sufficient to improve tissue generation. By contrast, reconstruction was better in the hNSC Implants group, and represented 32.5% (31.5; 39.0) (*p* < 0.05), showing that this combination stimulated tissue reconstruction around the injured area.

### Immunohistological characterization of brain tissue regeneration

**Fig. 4:**
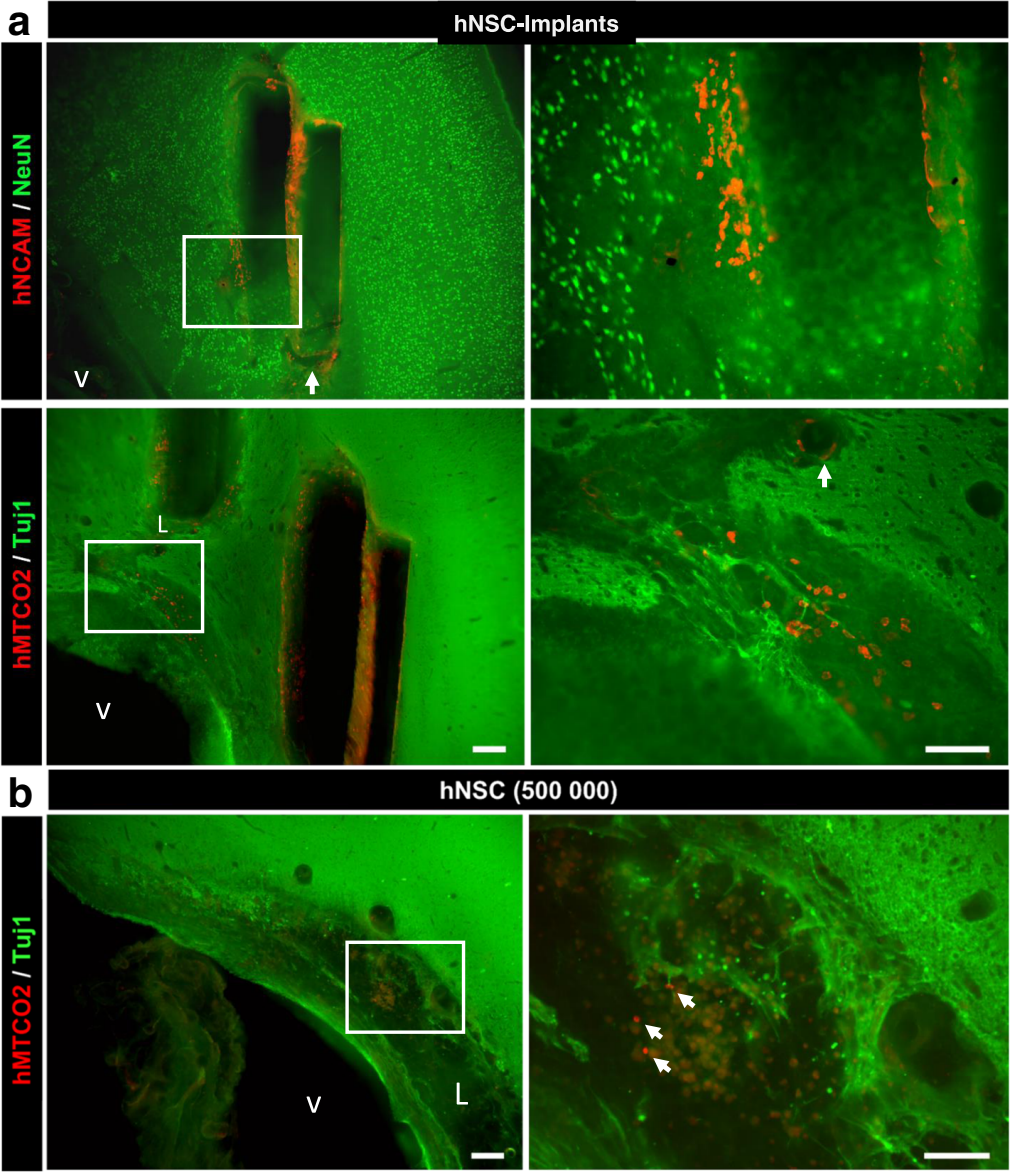
**a** hNSC location and survival around neuro-implants 3 months after graft. hNSCs were identified by two specific human markers (red), hMTCO2 or hNCAM, in combination with a marker (green) of immature (Tuj-1) or mature (NeuN) neurons. Low magnification on the left and higher magnification, corresponding to the area in the white square, on the right. White arrows indicate blood vessels (Axiozoom images, scale bar: 100 μm). **b** hNSC detection 3 months after a graft of hNSCs alone. hNSCs identified in red by hMTCO2 and in green by Tuj-1. Low magnification on the left and higher magnification, corresponding to the area in the white square, on the right. White arrows indicate rare surviving hNSCs among a cluster of non-bright fluorescent cells corresponding certainly to non-alive cells (Axiozoom images, scale bar: 100 μm). V ventricle, L lesion, hNSC human neural stem cell, hMTCO2 human mitochondria marker (cytochrome c oxidase II), hNCAM human neural cell adhesion molecules, Tuj1 neuron-specific class III beta-tubulin, NeuN neuronal nuclear antigen

Examination of all stained sections from implanted lesioned animals with specific human markers revealed long-term survival of hNSCs (Fig. 4a), compared with the poor survival observed following hNSC transplantation (Fig. 4b). Indeed, fewer than 10 surviving cells were found in the hNSCs group and no reconstructed tissue was seen, thus this group was not fully explored in this study. In the hNSC Implants group, at 3 months post lesion, hNCAM and hMTCO2-immunoreactive cells were numerous, both near the implantation site and in the peri-lesional host tissue (Fig. 4a, top). No solid tumour-like growth was observed with the grafted cells judging from the human immunostaining. When hNSCs were co-grafted with implants they spread from the implants to the reconstructed tissues and the close peri-lesional host tissues, sometimes over quite long distances (see later), often along blood vessels (Fig. 4a, white arrows). Furthermore, they were found to be intermingled with newly generated Tuj1-positive neurons from the host (Fig. 4a, bottom).

#### Neuronal fate and host–scaffold interactions

**Fig. 5:**
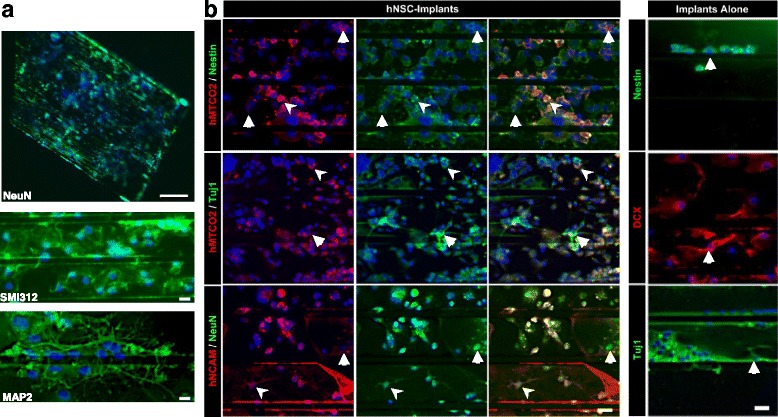
Immunofluorescence staining of hNSCs on neuro-implants 3 months after engraftment. **a** Typical distribution of hNSCs and their neuritic network on the PDMS scaffold stained with the neuronal markers (green) NeuN (top), SMI312 (middle) and MAP2 (bottom). Scale bars: 500 and 20 μm. **b** Detection of cell differentiation after 3 months on implants pre-seeded with hNSCs (hNSC Implants group) or on their own (Implants Alone group). Single or double staining was performed as indicated, with the human markers hMTCO2 or hNCAM (red) and markers for immature (nestin and Tuj1 in green and DCX in red) and mature (NeuN in green) neurons. Nuclei counterstained with DAPI. White arrowheads indicate marker co-localization and arrows indicate single marker expression. Axiozoom images, scale bar: 20μm. hNSC human neural stem cell, hMTCO2 human mitochondria marker (cytochrome c oxidase II), hNCAM human neural cell adhesion molecules, Tuj1 neuron-specific class III beta-tubulin, MAP2 microtubule associated protein 2, NeuN neuronal nuclear antigen, DCX doublecortin

The fate of the grafted cells was first studied on the implants, by direct immunostaining on the neuro-implants retrieved after rat sacrifice (Fig. 5). Considerable cell density was still found on implants at 3 months, homogeneously distributed after insertion in the lesion (Fig. 5a). Interestingly, the cells were in the grooves and expressed mature neuronal markers such as NeuN, MAP2 and SMI312. Their axonal network was well established along the straight microchannels (SMI312; Fig. 5a) as has previously been observed in vitro [28].

The double-staining study, using specific human markers, confirmed the presence of grafted cells, and revealed that cells from the host were also present on the implants above all when hNSCs had been pre-seeded (Fig. 5b). Some hNSCs were still expressing immature neural markers after 3 months. The proportion of hNCAM-positive and nestin-positive double-labelled cells was 51%. A total of 28% of hNSCs were also Tuj1-positive and, interestingly, 27% had been able to mature and express neuronal markers as NeuN. Most of the host cells observed on the implants expressed immature markers (nestin, DCX and Tuj1; Fig. 5b). However, a few expressed MAP2 (Fig. 5a), demonstrating a process of maturation, especially when hNSCs were on the implants as well. Differentiation of both hNSCs and progenitor cells from the host were observed on the PDMS scaffolds, emphasizing the positive interaction with the biomaterial.

### Short-distance migration of grafted cells

**Fig. 6:**
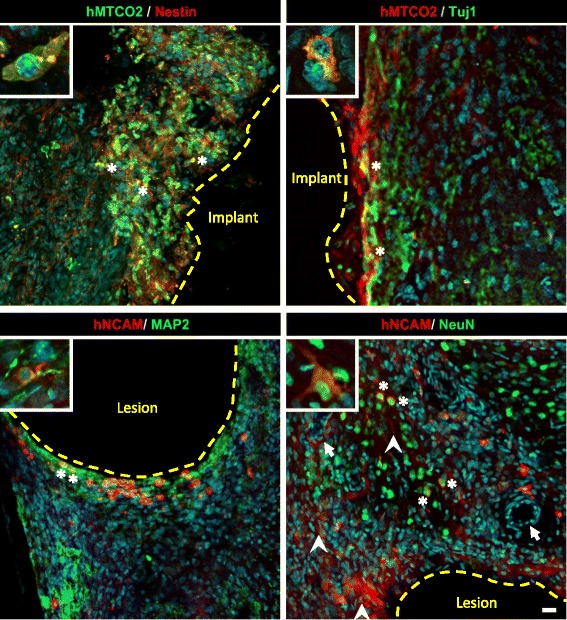
Immunofluorescence characterization of hNSC differentiation in reconstructed and host peri-lesional tissues. Double staining performed as indicated, with human markers (hMTCO2 or hNCAM) and markers for immature (nestin and Tuj1) and mature (MAP2 and NeuN) neurons. Representative double staining shown at higher magnification in the insets. Nuclei counterstained with DAPI. White arrows indicate blood vessels, white arrowheads indicate human fibres and asterisks localize typical double staining. Confocal images, scale bar: 20 μm. hMTCO2 human mitochondria marker (cytochrome c oxidase II), hNCAM human neural cell adhesion molecules, Tuj1 neuron-specific class III beta-tubulin, MAP2 microtubule associated protein 2, NeuN neuronal nuclear antigen

Many grafted hNSCs were also found migrating to and integrating with the host tissue, close to implant location, in the reconstructed and peri-lesional tissues (Fig. 6, white arrowhead: hNCAM-positive fibres). A total of 33% of these cells remained immature (nestin-positive cells), even at 3 months, but 32% were engaged in a neuronal differentiation pathway (Tuj1-poasitive; Fig. 6, top). A large proportion of the cells that succeeded in colonizing the host tissue became mature neurons (22% MAP2-positive and 26% NeuN-positive; Fig. 6, bottom).

#### Specific and long-distance migration of grafted cells

**Fig. 7:**
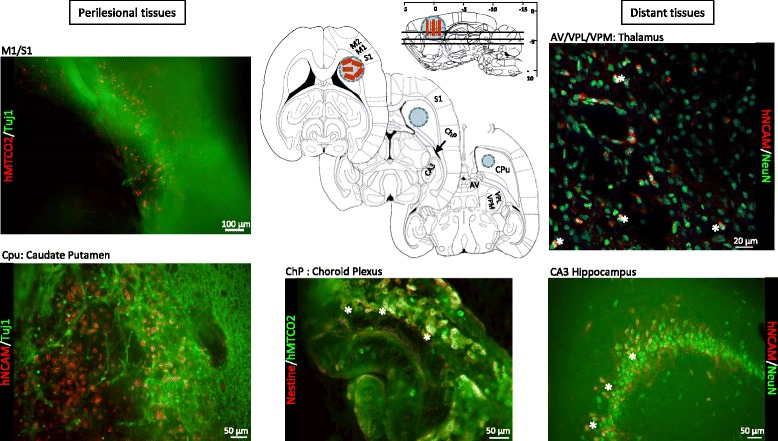
Localization of migrant hNSCs grafted in the brain on implants, detected by immunofluorescence staining. Identification of the migration area on three axial slices (Paxinos and Watson atlas [32]) at Bregma coordinates: –3.60 mm, –4.60 mm and –5.82 mm. Lesion indicated by blue circle on slices of the whole brain, and red rectangles represent implants. hNSCs identified by the specific human markers (red, hMTCO2 and hNCAM), in combination with different markers of maturation (green, nestin, Tuj1 or NeuN). Names of structures indicated at the level where hNSCs were retrieved. AV antero-ventral thalamic nucleus, CA3 CA3 field of hippocampus, ChP choroid plexus, CPu caudate/putamen, M1 primary motor cortex, M2 secondary motor cortex, S1 primary somatosensory cortex, VPM ventral posteromedial thalamic nucleus, VPL ventral posterolateral thalamic nucleus, hMTCO2 human mitochondria marker (cytochrome c oxidase II), hNCAM human neural cell adhesion molecules, NeuN neuronal nuclear antigen

Finally, in the host tissue, some grafted cells were detected both in the peri-lesional area, notably in deep layers of peri-lesional motor cortices M1, M2, S1-jaw and S1-upper limb (*n* = 2; Fig. 7), and far away from the implantation/lesion site, the precise location depending on the animal. In the rats studied by histology (*n* = 5), a large proportion of hNSCs was found deep in the lesion, in the caudate-putamen area at the dorso-ventral coordinates (–4.60 to –5.80 mm from Bregma) (Fig. 7). They were also detected sticking to the choroid plexus of the lateral ventricle (*n* = 5). In these areas, the cells retained a stemness characteristic, as they expressed the immature marker nestin. Unexpectedly, in some animals, a few migrant grafted cells were found deep below the lesion, in other brain structures such as the thalamus (antero-ventral (AV)/ventral posterolateral (VPL)/ventral posteromedial nucleus (VPM); *n* = 2) and the caudal hippocampus, 5 mm behind Bregma, in the CA3 layer (*n* = 2). In these host functional structures, the cells were integrated with the tissue as new mature neurons (NeuN-positive). hNSCs therefore have an integration potential in the host tissue.

### Inflammation

**Fig. 8:**
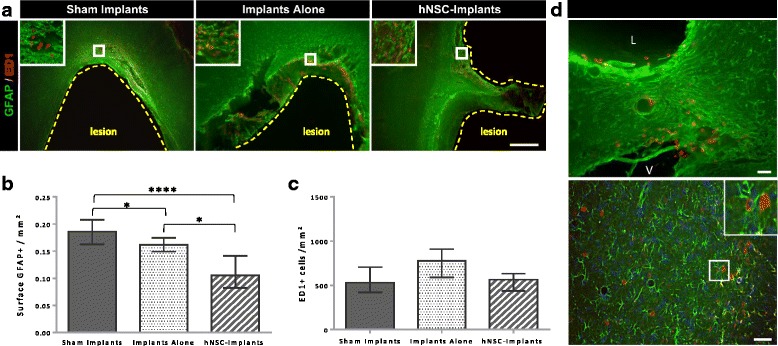
Assessment of astrocyte and inflammation reaction near cystic cavity and implantation site. **a** Typical appearance of fluorescent immunostaining for GFAP, the astroglial marker (green) and ED1, a cellular marker specific to activated microglia, monocytes and macrophages (red) on Axiozoom images. White squares indicate higher magnification area of insets. Scale bar: 500 μm. **b** Immunostaining quantification of the astroglial signal: data expressed as the surface of GFAP-positive signal/mm^2^ of tissue analysed in each group (*n* = 5 per group). **c** Number of ED1 immunoreactive cells/mm^2^ of tissue analysed (*n* = 5 per group). Data represent median ± interquartile range. Values analysed with the Kruskal–Wallis test followed by corrected Dunn’s test. *p < 0.05. ***p < 0.0001. **d** Immunofluorescence detection of host or human astrocytes in the tissue of hNSC Implants rats 3 months post graft. Astrocytes (GFAP) in green and hNSCs (hNCAM) in red. Representative double staining shown at higher magnification. Scale bar: 50 μm. L lesion, V ventricle, hNSC human neural stem cell, GFAP glial fibrillary acidic protein, hNCAM human neural cell adhesion molecules

An inflammatory and astroglial reaction was visible in all groups 3 months after induction of the lesion, and formed a glial scar around the lesion (ED1-positive and GFAP-positive cells; Fig. 8a). In the Sham Implants group, 18.6% of the analysed tissue was GFAP-positive (Fig. 8b). The astroglial reaction was also detectable, albeit less intense, when the animals received implants without hNSCs (16.1% of the analysed tissue, *p* < 0.05), suggesting good biocompatibility of PDMS implants. A fraction of the seeded hNSCs were able to differentiate into astrocytes (hNCAM-positive; Fig. 8d), but there were too few of them to contribute much to glial scar formation. In any case, the combination of implants with neuronal cells prevented glial scar formation and considerably reduced the astroglial reaction (10.5% of analysed tissue) compared with the Sham Implants (*p* < 0.0001) and Implants Alone (*p* < 0.05) groups. Regarding the microglial activation initially caused by the lesion, PDMS implants with or without grafted cells did not seem to have any impact (Fig. 8c).

## Discussion

These results demonstrate for the first time an improvement in motor recovery following engraftment of microstructured scaffolds with pre-seeded primary adult hNSCs, compared with other treatments such as direct cell graft or scaffold-alone insertion. This study confirms the results obtained previously with the neuronal cell line hNT2 pre-seeded on the same scaffolds [29], and provides new insights into the underlying mechanisms behind this behavioural improvement. Both grafted and host cells were found on implants at 3 months, despite the quite hostile environment of the lesion. Implant insertion stimulated tissue reconstruction in the injured area, notably in the presence of hNSCs, which could differentiate into mature neurons. These supported host neurogenesis, and limited atrophy and glial reaction. Finally, some hNSCs showed targeted migration and neuronal maturation abilities in cerebral structures close to and away from the implantation site.

### Multiple bio-implant effects on tissue reorganization and motor recovery

Olstorn et al*.* [24] described the tremendous potential of adult hNSCs to migrate towards the lesion and to mature into neurons. To our knowledge, however, our study was the first time that adult hNSCs had been grafted intracerebrally in combination with biomaterials. The beneficial effect on behaviour was more significant and more prominent than a direct graft of five times more cells. Even if cells taken from adult brains have limited proliferation ability, they can survive on PDMS without any added growth factors or nutrients released by a degradable material. One reason for this survival is that the 3D microstructures of the device protect the cells during implantation, and the 3-day pre-seeding of the cells certainly limits the latency period that is often observed after a direct cell graft [29]. Therefore, this engraftment had led to tissue reconstruction filling 32.5% of the lesion. The effect was better than that of other approaches, which have achieved maximum reconstruction of around 5% [35, 36], but not as good as the effect of Matrigel™ mixed with cells, which reconstructed around 50% of the lesion [21]. Owing to its undefined composition, however, Matrigel™ is not suitable for clinical applications [37].

In the present study, we also showed that the presence of PDMS implants had further beneficial effects. First, the scaffolds restricted hemispheric atrophy by sustaining tissue and limited dilation of the ipsilesional lateral ventricle. The brain/cerebral morphology was therefore preserved. This effect has also been observed in another study, 60 days after lesion of the cerebral cortex [38]. Maintaining the integrity of the brain shape is undoubtedly an important process, as it certainly avoids damage aggravation. Peri-lesional areas and callosal fibres are key structures for natural plasticity, which promotes particularly forelimb sensorimotor functional recovery [39]. Second, implant insertion reduced the glial reaction, especially when hNSCs were present. Moreover, the presence of co-grafted hNSCs has been shown to have a tendency to reduce the inflammatory reaction, as they downregulate the expression of chemokines such as TNF-α, IL-6 and IL-1β [20] and make the environment less hostile. The establishment of a glial scar after a stroke is a normal process, and has several benefits, including protecting neural cells and restricting the spread of inflammation [40, 41]. By contrast, a very large glial scar acts as a barrier, precludes passage of chemoattractant signals and, consequently, of cells and thus prevents all possibility of reconstruction [42–44]. In our study, astroglial activation was significantly less intense, and there was a tiny glial scar around the lesion cavity in the hNSC Implants group, which may favour reconstruction.

### Bidirectional interactions with the host tissue and neuronal maturation

In the present study, cells from the host tissue could be detected on the biomaterial 3 months after implantation in the lesion. This attraction has been observed with scaffolds of different compositions, notably PLA [45], PLGA [15, 46], gelatin–siloxane hybrid [38] and collagen [35]. The biomaterial in our study may have favoured hNSC differentiation in the brain by mimicking the extracellular matrix. It also acted as a scaffold for endogenous neuroblasts that had migrated up to the infarct area. Their maturation into neurons was better in the presence of hNSCs than on implants alone. This neurotrophic effect has already been described [47, 48], and grafted cells have been shown to be capable of making new connections with host tissue cells [49]. Consequently, both populations are perhaps responsible for the effect in functional recovery.

Grafted hNSCs were able to mature on implants and around the lesion site. Nevertheless, some of the grafted hNSCs around the lesion site remained immature. Even 3 months post lesion, the mechanisms of endogenous neurogenesis, migration and reconstruction still seemed to be in progress, possibly suggesting that recovery could improve with time. Our serial behavioural assessment allowed us to show that a greater sensorimotor improvement occurred not immediately after the implantation but 2 months post lesion, which might be explained by a reconstruction effect rather than just a trophic effect.

### Short-distance and long-distance migration

Some hNSCs migrated from the implants to the surrounding host tissue. They were often found close to blood vessels, even at 3 months post lesion, in agreement with other studies demonstrating that the vascular network can guide stem-cell migration [50, 51]. Thored et al. [52] found neuroblasts migrating towards the ischaemic area, in the vicinity of blood vessels, up to 16 weeks post infarct. Moreover, after migration, endothelial cells stimulate self-renewal and promote the maturation of NSCs in the tissue [53, 54]. This could explain our finding that a subset of co-grafted hNSCs succeeded in maturing around the lesion and in the host tissue. Another part of grafted hNSCs stayed in an immature or quiescent state in the choroid plexus, which is a component of the adult SVZ niche that could support their proliferation [55].

A lesion of the primary motor cortex induces a motor deficit by degenerating fibres of the corticospinal tract (CST), and creates secondary degeneration, indicated by the presence of pyknotic nuclei, mostly in thalamic motor nuclei (VPL, VPM). Monocyte chemoattractant protein-1 (MCP-1) is a critical molecule in the regulation of thalamic retrograde neuronal degeneration [56]. The thalamus is involved in the integration of sensory afferents and receives cortical motor efferent signals, playing a major role in the sensory motor loop. In our study, neuro-implants were inserted in the dorso-ventral axis to promote restoration of the sensorimotor loop. Some grafted hNSCs were able to migrate and integrate these secondary degenerated thalamic nuclei and become mature neurons (NeuN-positive cells), whereas they usually remain at the injection site in a healthy brain [49]. The implant position cannot alone explain migration to the thalamus. Chemotactic molecules may attract cells. The molecular mechanisms involved in directing the new neurons to the damaged areas revealed the role of blood vessels (endothelial cells) and inflammatory cells (reactive astrocyte and activated microglia), involving well-described factors such as stromal cell-derived factor 1 (SDF-1), BDNF, MCP-1, osteopontin and VEGF [7, 57]. Gaillard et al. [58] showed that connections can be established between a transplant of embryonic cortical neurons in the primary motor cortex and thalamus. These cell migrations/integrations and the design of neuro-implants can provide advantages to restore the CST and corticothalamic tracts, and could explain the enhanced motor strength with hNSC implants. It should be emphasized that migrant cells were mainly found in the ipsilesional hemisphere. Few hNSCs have migrated and integrated the caudal hippocampus (CA3). As far as we are aware, this is the first time that intracortically grafted cells were observed to have migrated to the caudal hippocampus, a few millimetres from the lesion. This could bring new assets for motor recovery, especially for post-lesional motor relearning, as the hippocampus has been found to be involved in motor learning [59].

## Conclusions

The combined insertion of hNSCs and implants may help motor recovery. The role of this association is multiple. Implants have sustained cerebral tissue, allowing the preservation of adjacent cortical areas which could be involved in natural plasticity. The presence of implants permitted the survival and maturation of both grafted hNSCs and endogenous neuroblasts, favouring tissue reconstruction. Moreover, the reduction of astroglial reaction has led to better bi-directional exchange between the implants and the host tissue. Tissue reconstruction and neuroblast maturation seem still ongoing 3 months post injury. This therapeutic strategy with long-lasting bio-implants seems to give an advantage over too rapidly biodegradable biomaterials regarding cell viability [60] and brings new hope for future applications.

## Abbreviations

AV: Antero-ventral thalamic nucleus
DCX: Doublecortin
GFAP: Glial fibrillary acidic protein
hMTCO2: Human mitochondria marker (cytochrome c oxidase II)
hNCAM: Human neural cell adhesion molecules
hNSC: Human neural stem cell
M1: Primary motor cortex
MAP2: Microtubule associated protein 2
NeuN: Neuronal nuclear antigen
NGF: Neural growth factor
PDMS: Polydimethylsiloxane
SMI312: Anti-neurofilament marker
Tuj1: Neuron-specific class III beta-tubulin
VPL: Ventral posterolateral thalamic nucleus
VPM: Ventral posteromedial thalamic nucleus

## References

[1] Riquelme PA, Drapeau E, Doetsch F (2008). Brain micro-ecologies: neural stem cell niches in the adult mammalian brain. Philos Trans R Soc B Biol Sci.

[2] Ma DK, Bonaguidi MA, Ming G, Song H (2009). Adult neural stem cells in the mammalian central nervous system. Cell Res.

[3] Gross CG (2000). Neurogenesis in the adult brain: death of a dogma. Nat Rev Neurosci.

[4] Doetsch F (2003). The glial identity of neural stem cells. Nat Neurosci.

[5] Nam H, Lee K-H, Nam D-H, Joo KM (2015). Adult human neural stem cell therapeutics: current developmental status and prospect. World J Stem Cells.

[6] Thored P, Arvidsson A, Cacci E, Ahlenius H, Kallur T, Darsalia V (2006). Persistent production of neurons from adult brain stem cells during recovery after stroke. Stem Cells.

[7] Lindvall O, Kokaia Z (2015). Neurogenesis following stroke affecting the adult brain. Cold Spring Harb Perspect Biol.

[8] Zhang R, Zhang Z, Wang L, Wang Y, Gousev A, Zhang L (2004). Activated neural stem cells contribute to stroke-induced neurogenesis and neuroblast migration toward the infarct boundary in adult rats. J Cereb Blood Flow Metab.

[9] Osman AM, Porritt MJ, Nilsson M, Kuhn HG (2011). Long-term stimulation of neural progenitor cell migration after cortical ischemia in mice. Stroke.

[10] Arvidsson A, Collin T, Kirik D, Kokaia Z, Lindvall O (2002). Neuronal replacement from endogenous precursors in the adult brain after stroke. Nat Med.

[11] Parent JM, Vexler ZS, Gong C, Derugin N, Ferriero DM (2002). Rat forebrain neurogenesis and striatal neuron replacement after focal stroke. Ann Neurol.

[12] Saha B, Peron S, Murray K, Jaber M, Gaillard A (2013). Cortical lesion stimulates adult subventricular zone neural progenitor cell proliferation and migration to the site of injury. Stem Cell Res.

[13] Hicks C, Stevanato L, Stroemer RP, Tang E, Richardson S, Sinden JD (2013). In vivo and in vitro characterization of the angiogenic effect of CTX0E03 human neural stem cells. Cell Transplant.

[14] Michelsen KA, Acosta-Verdugo S, Benoit-Marand M, Espuny-Camacho I, Gaspard N, Saha B (2015). Area-specific reestablishment of damaged circuits in the adult cerebral cortex by cortical neurons derived from mouse embryonic stem cells. Neuron.

[15] Quittet M-S, Touzani O, Sindji L, Cayon J, Fillesoye F, Toutain J (2015). Effects of mesenchymal stem cell therapy, in association with pharmacologically active microcarriers releasing VEGF, in an ischaemic stroke model in the rat. Acta Biomater.

[16] Braun H, Günther-Kern A, Reymann K, Onteniente B (2012). Neuronal differentiation of human iPS-cells in a rat cortical primary culture. Acta Neurobiol Exp.

[17] Moe MC, Varghese M, Danilov AI, Westerlund U, Ramm-Pettersen J, Brundin L (2005). Multipotent progenitor cells from the adult human brain: neurophysiological differentiation to mature neurons. Brain.

[18] Vaysse L, Labie C, Canolle B, Jozan S, Béduer A, Arnauduc F (2012). Adult human progenitor cells from the temporal lobe: another source of neuronal cells. Brain Inj.

[19] Westerlund U, Svensson M, Moe MC, Varghese M, Gustavsson B, Wallstedt L, et al. Endoscopically harvested stem cells: a putative method in future autotransplantation. Neurosurgery. 2005;55:779–84. 16239892

[20] Huang L, Wong S, Snyder EY, Hamblin MH, Lee J-P. Human neural stem cells rapidly ameliorate symptomatic inflammation in early-stage ischemic-reperfusion cerebral injury. Stem Cell Res. Ther. 2014;5:129. 10.1186/scrt519 PMC4445985 25418536

[21] Jin K, Mao X, Xie L, Galvan V, Lai B, Wang Y (2010). Transplantation of human neural precursor cells in Matrigel scaffolding improves outcome from focal cerebral ischemia after delayed postischemic treatment in rats. J Cereb Blood Flow Metab Off J Int Soc Cereb Blood Flow Metab.

[22] Guan J, Zhu Z, Zhao RC, Xiao Z, Wu C, Han Q (2013). Transplantation of human mesenchymal stem cells loaded on collagen scaffolds for the treatment of traumatic brain injury in rats. Biomaterials.

[23] André EM, Passirani C, Seijo B, Sanchez A, Montero-Menei CN (2016). Nano and microcarriers to improve stem cell behaviour for neuroregenerative medicine strategies: application to Huntington’s disease. Biomaterials.

[24] Olstorn H, Varghese M, Murrell W, Moe MC, Langmoen IA (2011). Predifferentiated brain-derived adult human progenitor cells migrate toward ischemia after transplantation to the adult rat brain. Neurosurgery.

[25] Nakaji-Hirabayashi T, Kato K, Iwata H (2009). Hyaluronic acid hydrogel loaded with genetically-engineered brain-derived neurotrophic factor as a neural cell carrier. Biomaterials.

[26] Elias PZ, Spector M (2012). Implantation of a collagen scaffold seeded with adult rat hippocampal progenitors in a rat model of penetrating brain injury. J Neurosci Methods.

[27] Vaysse L, Conchou F, Demain B, Davoust C, Plas B, Ruggieri C (2015). Strength and fine dexterity recovery profiles after a primary motor cortex insult and effect of a neuronal cell graft. Behav Neurosci.

[28] Béduer A, Vieu C, Arnauduc F, Sol J-C, Loubinoux I, Vaysse L (2012). Engineering of adult human neural stem cells differentiation through surface micropatterning. Biomaterials.

[29] Vaysse L, Beduer A, Sol JC, Vieu C, Loubinoux I (2015). Micropatterned bioimplant with guided neuronal cells to promote tissue reconstruction and improve functional recovery after primary motor cortex insult. Biomaterials.

[30] Béduer A, Vaysse L, Flahaut E, Seichepine F, Loubinoux I, Vieu C (2011). Multi-scale engineering for neuronal cell growth and differentiation. Microelectron Eng.

[31] Hassler C, Boretius T, Stieglitz T (2011). Polymers for neural implants. J Polym Sci Part B Polym Phys.

[32] Paxinos G, Watson C. The Rat Brain in Stereotaxic Coordinates. 4nd ed. San Diego: Academic Press; 1998.

[33] Baayen RH, Davidson DJ, Bates DM (2008). Mixed-effects modeling with crossed random effects for subjects and items. J Mem Lang.

[34] Pinheiro JC, Bates DM. Theory and Computational Methods for Linear Mixed-Effects Models. In: Mixed-Effects Models in S and S-PLUS. Statistics and Computing. New York: Springer; 2000. p. 3-56.

[35] Lu D, Mahmood A, Qu C, Hong X, Kaplan D, Chopp M (2007). Collagen scaffolds populated with human marrow stromal cells reduce lesion volume and improve functional outcome after traumatic brain injury. Neurosurgery.

[36] Qu C, Xiong Y, Mahmood A, Kaplan DL, Goussev A, Ning R (2009). Treatment of traumatic brain injury in mice with bone marrow stromal cell-impregnated collagen scaffolds. J Neurosurg.

[37] Wong Po Foo CTS, Lee JS, Mulyasasmita W, Parisi-Amon A, Heilshorn SC (2009). Two-component protein-engineered physical hydrogels for cell encapsulation. Proc Natl Acad Sci U S A.

[38] Deguchi K, Tsuru K, Hayashi T, Takaishi M, Nagahara M, Nagotani S (2006). Implantation of a new porous gelatin–siloxane hybrid into a brain lesion as a potential scaffold for tissue regeneration. J Cereb Blood Flow Metab.

[39] Starkey ML, Bleul C, Zorner B, Lindau NT, Mueggler T, Rudin M (2012). Back seat driving: hindlimb corticospinal neurons assume forelimb control following ischaemic stroke. Brain.

[40] Anderson MA, Burda JE, Ren Y, Ao Y, O’Shea TM, Kawaguchi R (2016). Astrocyte scar formation aids central nervous system axon regeneration. Nature.

[41] Burda JE, Sofroniew MV (2014). Reactive gliosis and the multicellular response to CNS damage and disease. Neuron.

[42] Menet V, Prieto M, Privat A, Ribotta MG (2003). Axonal plasticity and functional recovery after spinal cord injury in mice deficient in both glial fibrillary acidic protein and vimentin genes. Proc Natl Acad Sci U S A.

[43] Silver J, Miller JH (2004). Regeneration beyond the glial scar. Nat Rev Neurosci.

[44] Peruzzotti-Jametti L, Donegá M, Giusto E, Mallucci G, Marchetti B, Pluchino S. The role of the immune system in central nervous system plasticity after acute injury. Neuroscience. 2014;44:210–21. 10.1016/j.neuroscience.2014.04.036 PMC4167877 24785677

[45] Álvarez Z, Castaño O, Castells AA, Mateos-Timoneda MA, Planell JA, Engel E (2014). Neurogenesis and vascularization of the damaged brain using a lactate-releasing biomimetic scaffold. Biomaterials.

[46] Bible E, Chau DYS, Alexander MR, Price J, Shakesheff KM, Modo M (2009). The support of neural stem cells transplanted into stroke-induced brain cavities by PLGA particles. Biomaterials.

[47] Cossetti C, Alfaro-Cervello C, Donegà M, Tyzack G, Pluchino S (2012). New perspectives of tissue remodelling with neural stem and progenitor cell-based therapies. Cell Tissue Res.

[48] Lu P, Jones LL, Snyder EY, Tuszynski MH (2003). Neural stem cells constitutively secrete neurotrophic factors and promote extensive host axonal growth after spinal cord injury. Exp Neurol.

[49] Kelly S, Bliss TM, Shah AK, Sun GH, Ma M, Foo WC (2004). Transplanted human fetal neural stem cells survive, migrate, and differentiate in ischemic rat cerebral cortex. Proc Natl Acad Sci U S A.

[50] Koutsakis C, Kazanis I. How necessary is the vasculature in the life of neural stem and progenitor cells? Evidence from evolution, development and the adult nervous system. Front Cell Neurosci. 2016;10:35. doi: 10.3389/fncel.2016.00035. 10.3389/fncel.2016.00035 PMC4754404 26909025

[51] Kojima T, Hirota Y, Ema M, Takahashi S, Miyoshi I, Okano H (2010). Subventricular zone-derived neural progenitor cells migrate along a blood vessel scaffold toward the post-stroke striatum. Stem Cells.

[52] Thored P, Wood J, Arvidsson A, Cammenga J, Kokaia Z, Lindvall O (2007). Long-term neuroblast migration along blood vessels in an area with transient angiogenesis and increased vascularization after stroke. Stroke.

[53] Ohab JJ, Fleming S, Blesch A, Carmichael ST (2006). A neurovascular niche for neurogenesis after stroke. J Neurosci Off J Soc Neurosci.

[54] Shen Q, Goderie SK, Jin L, Karanth N, Sun Y, Abramova N (2004). Endothelial cells stimulate self-renewal and expand neurogenesis of neural stem cells. Science.

[55] Silva-Vargas V, Maldonado-Soto AR, Mizrak D, Codega P, Doetsch F (2016). Age-dependent niche signals from the choroid plexus regulate adult neural stem cells. Cell Stem Cell.

[56] Muessel MJ, Berman NE, Klein RM (2000). Early and specific expression of monocyte chemoattractant protein-1 in the thalamus induced by cortical injury. Brain Res.

[57] Wang Y, Deng Y, Zhou G-Q (2008). SDF-1α/CXCR4-mediated migration of systemically transplanted bone marrow stromal cells towards ischemic brain lesion in a rat model. Brain Res.

[58] Gaillard A, Prestoz L, Dumartin B, Cantereau A, Morel F, Roger M (2007). Reestablishment of damaged adult motor pathways by grafted embryonic cortical neurons. Nat Neurosci.

[59] Albouy G, Sterpenich V, Balteau E, Vandewalle G, Desseilles M, Dang-Vu T (2008). Both the hippocampus and striatum are involved in consolidation of motor sequence memory. Neuron.

[60] Wong FSY, Chan BP, Lo ACY (2014). Carriers in cell-based therapies for neurological disorders. Int J Mol Sci.

